# Unexpected malignancy at the time of hysterectomy performed for a benign indication: A retrospective review

**DOI:** 10.1371/journal.pone.0266338

**Published:** 2022-04-01

**Authors:** Cara G. Elliott, Ally Murji, John Matelski, Adebanke Bianca Adekola, Jessica Chrzanowski, Lindsay Shirreff

**Affiliations:** 1 Faculty of Medicine, University of Toronto, Toronto, Ontario, Canada; 2 Department of Obstetrics and Gynaecology, Mount Sinai Hospital, University of Toronto, Toronto, Ontario, Canada; 3 Department of Obstetrics and Gynaecology, University of Toronto, Toronto, Ontario, Canada; 4 Biostatistics Research Unit, Toronto General Hospital, University Health Network, Toronto, Ontario, Canada; Dipartimento di Scienze Mediche e Chirugiche (DIMEC), Orsola Hospital, ITALY

## Abstract

**Objective:**

To determine the proportion of patients undergoing hysterectomy for a benign indication who have unexpected malignancy (UM) on postoperative pathology and characterize the nature of UMs.

**Methods:**

This was a multi-center, retrospective study of patients undergoing hysterectomy for a benign indication from July 2016 to December 2019 at 7 Ontario, Canada hospitals (4 academic, 3 community). Hysterectomies for invasive placentation, malignant, and premalignant indications were excluded. Primary outcome was rate of unexpected malignancy as defined by the number of patients with malignancy on final pathology divided by the total number of hysterectomy cases. Data was extracted from health records and electronic charts. Patient, surgical, and surgeon characteristics were compared between benign and UM groups using bivariate methods. Associations between UM status and perioperative variables were assessed using bivariate logistic regression.

**Results:**

In the study period, 2779 hysterectomies were performed. UM incidence was 1.8% (51 malignancies/2779 cases), with one patient having two malignancies (total UMs = 52). The most common UM types were endometrial (27/52, 51.9%) and sarcoma (13/52, 25%). Patients with UM were older (57.2 ± 11.4 years vs. 52.8 ± 12.5 years, p = .015), had more previous laparotomies (2 (1.25, 2.0) vs. 1 (1.0, 1.0), p < .001), and higher BMI (29.7 ± 7.2 kg/m^2^ vs. 28.0 ± 5.9 kg/m^2^, p = .049) and ASA class (p < .028). Regarding surgical factors, patients with UM had more adhesions (p = .001), transfusions (p = .020), and blood loss (p = .006) compared to those with benign pathology. Patient characteristics most strongly associated with UM were age (OR 2.57, 95% CI 1.78–3.72, p < .001) and preoperative diagnosis of pelvic mass (OR 2.76, 95% CI 1.11–6.20, p = .019).

**Conclusion:**

Incidence of UM at hysterectomy for benign indication was 1.8%. Several perioperative variables are associated with an increased chance of UM.

## Introduction

Accurate preoperative diagnoses are essential to allow surgeons to counsel patients preoperatively and manage intraoperative planning [1,2]. When presumably benign tissue is excised at surgery but the pathologic diagnosis is unexpectedly malignant, a new patient management plan must be created. Surgeons and oncologists must determine whether their patient has been properly staged or undergone necessary intraoperative procedures. For instance, patients with endometrial cancer require bilateral salpingo-oophorectomy, which may not have been done concomitantly with hysterectomy if the malignancy was unexpected [3,4]. Surgeons must also examine if any intra-operative procedures may have inadvertently upstaged the malignancy [5]. For example, intraoperative rupture of a benign ovarian cyst that is unexpectantly malignant upstages ovarian cancer [6] and intra-abdominal morcellation of malignant uterine tissue may facilitate growth and metastasis of cancer cells [7].

There is little research exploring the rate of unexpected malignancy at the time of hysterectomy performed for benign indications. Small retrospective studies have estimated the incidence between 0.25% [8] to 0.4% [9], although a larger American study of 6360 hysterectomies reported the rate of unexpected malignancy to be 2.7% [10]. To our knowledge, there is no literature on long-term assessment of hysterectomy procedures over multiple consecutive years among a Canadian patient population. The objective of this large-scale, multi-site retrospective review was to determine the proportion of patients undergoing hysterectomy for benign indications who had unexpected malignancy (UM) on postoperative pathology and to characterize the nature of UMs. We also aimed to determine factors associated with unexpected malignancy discovered post-hysterectomy.

## Methods

We performed a multi-center retrospective study of all consecutive hysterectomy procedures at seven Ontario hospitals (4 academic, 3 community) between July 2016 and December 2019. Hysterectomies completed by gynecologic oncologists or those completed for a preoperative diagnosis of premalignant, malignant, invasive placentation, or uncertain diagnosis were excluded from the analysis. Data was extracted using a combination of health record coding (International Classification of Diseases, 10^th^ revision, ICD-10) and review of electronic medical records. Data from each site was entered into a central Research Electronic Capture (REDCap) registry. Data quality-assurance measures included real-time flagging of missing data and values outside pre-established ranges, random chart review of 10% of cases by an independent evaluator, and independent review and grading of each complication by two evaluators (research assistant and staff gynecologist). Research ethics board approval was obtained (20-0030-C).

### Outcomes

The exposure of interest was hysterectomies performed by gynecologists for benign indications. The primary outcome was the proportion of patients with unexpected malignancy at the time of hysterectomy performed for a benign indication. Unexpected malignancy was defined as a malignancy on final pathology report from a procedure without a malignant or premalignant (i.e. endometrial hyperplasia or cervical dysplasia) preoperative surgical indication. Incidence of unexpected malignancy was calculated by determining the number of patients with unexpected malignancy over the study period divided by the number of hysterectomy cases in our cohort.

Gynecologic malignancies were characterized by type including cervical cancer, uterine cancer (endometrial and sarcoma), fallopian tube cancer, and ovarian cancer. Non-gynecologic malignancies (i.e. appendiceal cancer) were also recorded.

### Covariates

We compared patients with unexpected malignancy to those without. We compared patient characteristics including age, body mass index (BMI), American Society of Anesthesiologist’s (ASA) class, previous abdominal or pelvic surgeries (laparotomy, laparoscopy or Caesarean section), and preoperative diagnoses. Surgical characteristics compared were route of hysterectomy (laparoscopic, vaginal, or abdominal), perioperative transfusion, estimated blood loss, presence of intraoperative endometriosis or adhesions, operative time, and uterine weight. We also recorded surgeon training and case volume. Surgeons were classified as either generalists (no additional training beyond residency) or fellowship-trained (Minimally Invasive Gynecologic Surgery (MIGS) or urogynecology/Pelvic Female Medicine and Reconstructive Surgery (FPMRS)). Fellowship training was considered additional training regardless of accreditation. Surgeon volume was defined as the mean number of hysterectomies performed over a 6-month period. Surgeons were considered low volume if they performed <6 cases per 6 months, average volume if 6 to 11 cases, and high volume if ≥ 12 cases. These cut-offs for surgical volume were informed by the literature [11].

### Statistics

Patient, surgical, and surgeon characteristics were compared between the benign and unexpected malignancy groups using standard bivariate methods (t-test, Wilcox rank sum test, chi-squared test, and Fisher’s exact test), as appropriate. Based on these findings, along with pragmatic and clinical considerations, we included several variables in the final multiple logistic regression model. It was anticipated the UM outcome was going to be infrequent, hence we were judicious in the covariates input into the model. Variables known to surgeons pre-operatively were selected as they would be most clinically meaningful with respect to preoperative counselling. We chose age, BMI, previous laparotomy, and selected preoperative diagnoses. We reported adjusted odds ratios (ORs), 95% confidence intervals (CIs), and Wald test p-values for the included variables. Patient age and BMI entered the model as continuous covariates and were scaled so the reported OR was for one standard deviation increase. ​The OR for previous laparotomy was expressed for a one-count increase in the number of previous laparotomies. We quantified the quality of model fit using the c-statistic with 95% confidence interval based on 1000 bootstrap samples. Alpha = .05 was adopted as the threshold for statistical significance. R version 3.6.2 software was used for the analysis [12].

## Results

**Fig 1:**
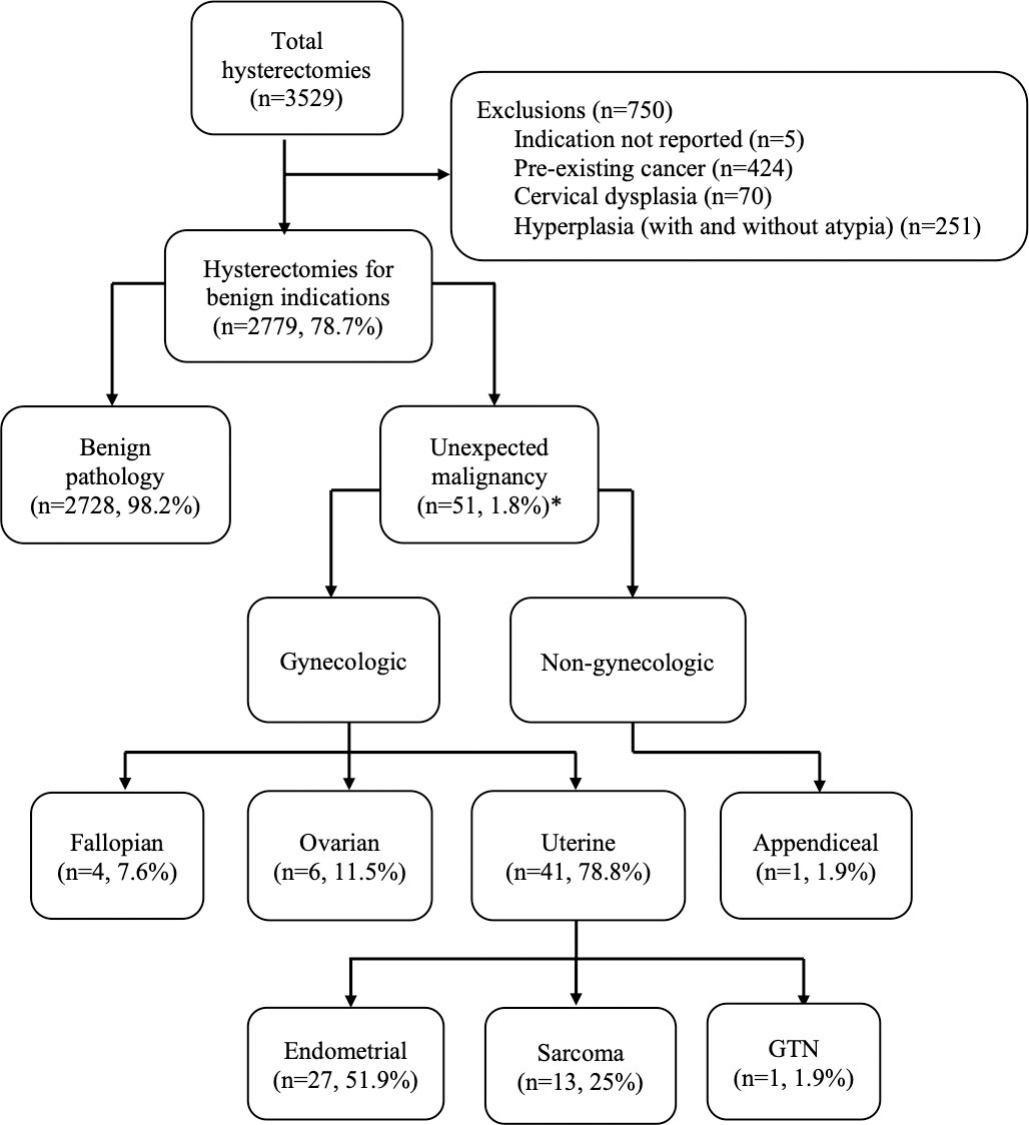
A flow diagram illustrating the number of unexpected malignancies on final pathology report for hysterectomy performed for benign indications. *51 patients had an unexpected malignancy with one patient having two malignancies (endometrial and appendiceal) (n = 52 total unexpected malignancies).

A total of 3529 hysterectomies were performed during the study period. After exclusions (n = 750), 2779 hysterectomies completed for benign preoperative diagnoses were analyzed. On final pathology, 2728 (98.2%) cases were reported as benign and 51 cases had at least one unexpectedly malignancy. One patient had two malignancies, including appendiceal, making the total number of unexpected malignancies in our cohort 52. The incidence of UM was 1.8% (51/2779). The most common gynecologic malignancy locations were uterine (endometrial (27/52, 51.9%), sarcoma (13/52, 25%) and gestational trophoblastic neoplasia (1/52, 1.9%)), ovarian (6/52, 11.5%) and fallopian tube (4/52, 7.6%). There were no occult cervical cancers (Fig 1).

Among patients with UM, the most common indications for surgery were fibroids (19/51, 37.2%) and abnormal uterine bleeding (16/51, 31.3%). Most patients with unexpected sarcoma had fibroids (12/13, 92.3%), while most with unexpected ovarian or fallopian tube cancer had pelvic mass as a preoperative diagnosis (6/10, 60%) (Table 1).

**Table 1:** Unexpected malignancy based on preoperative indication for surgery.

Surgical indication*	Unexpectant malignancy n (%)	Endometrial cancer n (%)	Ovarian, fallopian cancer n (%)	Sarcoma n (%)	GTN n (%)	Appendiceal n (%)
Abnormal uterine bleeding	16 (30.7)	12 (44.4)	2 (20)	2 (15.4)	0	0
Leiomyoma	19 (36.5)	6 (22.2)	1 (10)	12 (92.3)	0	0
Endometriosis/ adenomyosis/ pelvic pain/ dysmenorrhea	8 (15.4)	3 (11.1)	3 (30)	2 (15.3)	0	0
Prolapse/ voiding dysfunction/ stress urinary incontinence	9 (17.3)	6 (22.2)	1 (10)	2 (15.3)	0	0
Prophylactic/ risk reducing	3 (5.7)	1 (3.7)	1 (10)	0	0	1 (100)
Pelvic mass	13 (25)	3 (11.1)	6 (60)	3 (23.1)	1(100)	0
Other	4 (7.6)	4 (14.8)	0	0	0	0

*Patients may have had more than one indication for surgery.

GTN = gestational trophoblastic neoplasia.

Compared to patients who had confirmed benign pathology, patients with UM were older (57.2 ± 11.4 years vs. 52.8 ± 12.5 years, p = .015) and had more previous laparotomies (2 (1.25, 2.0) vs. 1 (1.0, 1.0), p < .001). Higher BMI (29.7 ± 7.2 kg/m^2^ vs. 28.0 ± 5.9 kg/m^2^, p = .049) and ASA class associated with UM (p < .028). (Table 2) With respect to surgical factors, patients with UM had more adhesions (p = .001), transfusions (p = .020), and blood loss (p = .006) compared to those with benign pathology. Prevalence of UM was higher among patients undergoing abdominal hysterectomy (45.1% vs. 20.8%, p = .003) and lower among those undergoing vaginal hysterectomy (13.7% vs. 38.6%, p = .001). Surgeon training and case volume were not associated with UM (Table 3).

**Table 2:** Preoperative patient characteristics of patients with benign and unexpected malignant pathology.

Characteristic	Total n = 2779	Benign n = 2728	Unexpectant malignancy n = 51	P-value*
Age, mean±SD	52.9 (12.5)	52.8 (12.5)	57.2 (11.4)	.015*
BMI (kg/m^2^), mean±SD	28.1 (5.9)	28.0 (5.9)	29.7 (7.2)	.049*
ASA Class, n (%)				
1	326 (11.7)	324 (11.9)	2 (3.9)	.028*
2	1481 (53.3)	1458 (53.4)	23 (45.1)	
≥3	972 (35.0)	946 (34.7)	26 (51.0)	
Surgical Indication, n (%)				
Abnormal uterine bleeding	603 (21.7)	587 (21.5)	16 (31.4)	.128**
Leiomyoma	995 (35.8)	976 (35.8)	19 (37.3)	.944
Endometriosis/ adenomyosis/ pelvic pain/ dysmenorrhea	499 (18.0)	491 (18.0)	8 (15.7)	.809*
Prolapse/ voiding dysfunction/ stress urinary incontinence	1068 (38.4)	1059 (38.8)	9 (17.6)	.003*
Prophylactic/ risk reducing	83 (3.0)	81 (3.0)	2 (3.9)	.664**
Pelvic mass	116 (4.2)	103 (3.8)	13 (35.5)	< .001**
Other	129 (4.6)	126 (4.6)	4 (7.8)	.297**
Previous Surgery (median, IQR)				
Laparotomy	1 (1.0, 1.0)	1 (1.0, 1.0)	2 (1.25, 2.0)	< .001***
n (%)	225 (8.1)	219 (8.0)	6 (11.8)	.300**
Laparoscopy	1 (1.0, 2.0)	1 (1.0, 2.0)	1 (1.0, 1.0)	.174***
n (%)	1123 (40.4)	1105 (40.5)	18 (35.3)	.544*
Caesarean section	1 (1.0, 2.0)	1 (1.0, 2.0)	1 (1.0, 1.5)	.293***
n (%)	499 (18.0)	488 (17.9)	11 (21.6)	.621*

SD = standard deviation

ASA = American Society of Anesthesiologists

BMI = body mass index

IQR = interquartile range

*T-test

**Fisher’s exact test

***Wilcox test.

**Table 3:** Surgical and surgeon characteristics of patients with benign and unexpected malignant pathology.

Characteristic	Total n = 2779	Benign n = 2728	Unexpectant malignancy n = 51	P-value*
**Surgical Characteristics**				
Concomitant, n (%)				
Endometriosis	369 (13.3)	363 (13.3)	6 (11.8)	.910*
Adhesions	863 (31.1)	836 (30.6)	27 (52.9)	.001*
Route of hysterectomy, n (%)				
Laparoscopic	1088 (39.2)	1067 (39.1)	21 (41.2)	.877**
Abdominal	591 (21.3)	568 (20.8)	23 (45.1)	< .001*
Vaginal	1059 (38.1)	1052 (38.6)	7 (13.7)	.001*
Uterine weight (median, IQR)	124 (62.2, 339.2)	124 (62, 333.1)	177 (70, 609)	.033***
0-500g, n (%)	2252 (82.0)	2216 (82.2)	36 (70.6)	.011*
501-1000g, n (%)	270 (9.8)	265 (9.8)	5 (9.8)	
>1000g, n (%)	225 (8.2)	215 (8.0)	10 (19.6)	
Transfusion, n (%)				
Perioperative	82 (3.0)	79 (2.9)	3 (5.9)	.189**
Number of units (median, IQR)	2 (1.0, 2.0)	2 (1.0, 2.0)	8 (5.0, 9.0)	.028***
Pre-op transfusion	18 (0.6)	18 (0.7)	0 (0)	1.000**
Number of units (median, IQR)	1.5 (1.0, 2.0)	1.5 (1.0, 2.0)	N/A	N/A***
Intra-op transfusion	32 (1.2)	29 (1.1)	3 (5.9)	.020**
Number of units (median, IQR)	1.5 (1.0, 2.0)	1 (1.0, 2.0)	8 (5.0, 9.0)	.017***
Post-op transfusion	40 (1.4)	40 (1.5)	0 (0.0)	1.000*
Number of units (median, IQR)	2 (1.0, 2.0)	2 (1.0, 2.0)	N/A	N/A***
Estimated blood loss, n (%)				
<250cc	1788 (68.9)	1763 (69.2)	25 (51.0)	.006*
250-500cc	658 (25.3)	641 (25.2)	17 (34.7)	
>500cc	150 (5.8)	143 (5.6)	7 (14.3)	
Operative time (min), n (%)	142 (100, 192)	142 (100, 191)	157 (104.5, 220)	.120***
**Surgeon Characteristics**				
Surgeon type, n (%)				
MIGS	829 (29.9)	812 (29.8)	17 (33.3)	.074*
Urogynecology	572 (20.6)	568 (20.8)	4 (7.8)	
Generalist	1376 (49.5)	1346 (49.4)	30 (58.8)	
Case volume (per yr), n (%)				
Low	988 (35.6)	967 (35.5)	21 (41.2)	.701*
Average	779 (28.1)	766 (28.1)	13 (35.5)	
High	1010 (36.4)	993 (36.4)	17 (33.3)	

SD = standard deviation; IQR = interquartile range; MIGS = Minimally Invasive Gynecologic Surgery

*T-test

**Fisher’s exact test

***Wilcox test.

The observed c-statistic for the multiple logistic regression model was .77 (95% bootstrap CI: .71 - .85), indicating good predictive accuracy of the model [13]. Patient characteristics most strongly associated with UM were age (OR 2.57, 95% CI 1.78–3.72, p < .001) and a preoperative diagnosis of pelvic mass (OR 2.76, 95% CI 1.11–6.20, p = .019). A preoperative diagnosis of prolapse, voiding dysfunction, or stress urinary incontinence was protective against UM (OR 0.12, 95% CI 0.05–0.3, p < .001) (Table 4).

**Table 4:** Logistic regression model between patient characteristics and UM.

Patient Characteristics	Odds ratio	95% CI	p-value^a^
Age	2.57	1.78–3.72	< .001
BMI	1.29	0.99–1.65	.051
Previous laparotomy	1.52	0.88–2.25	.056
Preoperative diagnosis			
Prolapse/ voiding dysfunction/ stress urinary incontinence	0.12	0.05–0.3	< .001
Prophylactic/ risk reducing	1.04	0.16–3.63	.960
Pelvic mass	2.76	1.11–6.20	.019

^a^Wald test.

BMI = body mass index.

## Discussion

In a large sample of 2779 hysterectomies performed for benign indications over three years, we found the incidence of unexpected malignancy was 1.8%. The most common sites of unexpected malignancy were uterine and ovarian and there were no unexpected cervical cancers. Patients with unexpected malignancy had significantly more adhesions, blood transfusions, and blood loss compared to patients who had benign pathology. Patient characteristics most predictive of unexpected malignancy were increasing age and preoperative diagnosis of pelvic mass. Seemingly protective against unexpected malignancy was a preoperative diagnosis of prolapse, voiding dysfunction, or stress urinary incontinence.

The incidence of unexpected malignancy in our study was comparable to previously published reports. Mahnert *et al*. identified an incidence of 2.7% in a cohort of 6360 benign hysterectomies completed in the United States, which included 11 patients with metastatic cancer and 11 patients with cervical cancer [10]. Interestingly, we reported no patients with occult cervical cancer or metastatic disease and our incidence of unexpected ovarian malignancy was lower than previous reports [14]. Hysterectomies in our cohort were completed within the publicly-funded Canadian healthcare system where patients have unrestricted access to robust cancer screening such as colonoscopy and pap smears and preoperative testing such as blood work, including tumor markers, and a variety of imaging modalities. Cervical and metastatic cancers may have been detected more readily, diverting these cases out of our cohort to a gynecologic oncologist for management, likely reflecting the strength and accessibility of robust cancer screening programs in our province.

Regarding unexpected endometrial cancer, our incidence (0.9%) was similar to that described by Mahnert *et al*. (1.02%) but higher than previous reports ranging from 0.13–0.45% [8,15–17]. Lower estimates from these earlier studies were likely due to extensive preoperative investigation whereby most patients underwent pap tests, ultrasound and/or endometrial sampling prior to hysterectomy [8,16]. While data on preoperative investigations was not collected in our study, we excluded patients with hyperplasia from our analysis and still found the incidence of unexpected endometrial cancer was similar to Mahnert *et al*., who included patients with hyperplasia in their cohort. Endometrial sampling may have been warranted but missed preoperatively among some patients in our population, highlighting the importance of careful preoperative assessment and endometrial sampling if risk factors are identified. These include elevated BMI and older age, both of which increased odds of unexpected malignancy in our study.

We determined a preoperative diagnosis of pelvic mass was associated with a higher chance of unexpected malignancy. In itself, this finding may not be surprising. However, it is interesting to quantify odds of UM among patients with this preoperative diagnosis and may help gynecologists in their preoperative counselling. This result also highlights the importance of accurately identifying adnexal masses as malignant before surgery to ensure procedures that may inadvertently upstage a malignancy, such as morcellation, are avoided and intraoperative staging opportunities are not missed [18]. Several diagnostic tests and algorithms exist to aid surgeons in clinical decision-making [19–22] with a recent review suggesting ultrasound-based prediction models International Ovarian Tumour Analysis LR2 [23] and Simple Rules [24] as the most sensitive and specific at achieving the highest diagnostic accuracy [25]. Application of such clinical prediction models and high-quality imaging may assist in patient counselling and surgical planning prior to hysterectomy.

Merits of this multi-center study included the large cohort of consecutive hysterectomies performed across Ontario in both academic and community hospitals. We used a robust dataset comprising of both administrative data and chart review. To our knowledge, this is the only Canadian study to report on the rates of unexpected malignancy at the time of benign hysterectomy and the first to assess surgeon characteristics.

Our results must be interpreted in the context of the study design. First, while preoperative testing was widely available to patients in our cohort, we did not assess presence or absence of preoperative evaluations such as endometrial sampling, tumor markers, or imaging reports. Depending on patients’ initial clinical presentation, foregoing various tests preoperatively may, in itself, have been predictive of malignancy. Second, we identified factors associated with unexpected malignancy, however, given the small sample we could not assess for factors associated with specific malignancies. Regardless, our findings can still help guide doctor-patient discussions and expectation-management with patients around risk of unexpected malignancy at the time of hysterectomy for benign indications. Last, we did not report disease outcomes among patients with unexpected malignancy. Further studies should be done to determine clinical course of patients with unexpected malignancy at hysterectomy to determine impact of this finding on prognosis, with a particular focus on patients with malignancy that was inadvertently upstaged.

## Conclusion

In summary, incidence of unexpected malignancy among patients having hysterectomy for a benign indication was 1.8%, with the most common cancer type being endometrial cancer and sarcoma. Several perioperative variables are associated with an increased chance of unexpected malignancy at the time of hysterectomy.
